# Exploring Muslim Communities’ Experiences and Barriers While Accessing Assisted Reproductive Technologies: A Scoping Review of International Literature

**DOI:** 10.1007/s10943-024-02056-x

**Published:** 2024-05-19

**Authors:** Kate Hammond, Nilab Hamidi

**Affiliations:** 1 Department of Social Work, The University of Melbourne, 161 Barry Street, Parkville, Melbourne, 3010 Australia; 2 Australian Muslim Women’s Centre for Human Rights, Melbourne, Australia

**Keywords:** ART, Infertility, Islam, Muslim, In vitro fertilization

## Abstract

This study adopted a scoping review methodology to analyze international literature on the barriers impacting Muslim couples’ access to equitable assisted reproductive technologies (ART). A total of 27 studies were included for review. Results show that Muslim communities face several barriers when accessing ART. These include cultural and religious barriers that impacted which aspects of ART couples were open to adopting, diminished quality of care due to low cultural/religious capacity of practitioners, as well as gendered norms which intersect with experiences of ART treatments. Further research, based in western countries, should be conducted to better understand how these contexts can support Muslim patients accessing ART.

## Introduction

### Infertility in Muslim Communities

Family and procreation are strongly encouraged in the Islamic faith. Marriage is often considered a prerequisite to building a family, as it performs the function of uniting the ‘two elements’ of humanity (men and women), and fosters procreation (Sachedina, 1990). The Qur’ān states of marriage and procreation, ‘And it is God who has given you spouses from amongst yourselves and through them He has given you children and grandchildren and provided you with good things…’ (*Qur’ān,* 16:72). Culturally, this religious belief is borne out in Muslim communities, where there is a strong emphasis on marriage, creating a family, and raising children (Al-Jayyousi et al., 2014).

A central component of Muslim family structures is transparency of lineage, particularly patrilineage. Establishing the patrilineal line of a child is paramount, as paternity has implications for other Islamic traditions and practices including inheritance, naming conventions, upholding *maharam* relationships (people with whom marriage is prohibited), and identifying daughters’ *wali* (male guardian) (Ayubi, 2021; Inhorn, 2006a; Monsoor, 2015). Such are the concerns surrounding the paternity of a child, that adoption, in the common sense of the word, is not permitted under Islamic law (Chaudhry, 2010). With such a strong emphasis on building a family, and limited options in terms of adoption, experiencing infertility can be a significant struggle for couples (Obeidat et al., 2014).

Infertility, as defined by the World Health Organization, is a condition characterized by the failure to achieve pregnancy after 12 months or more of regular unprotected sexual intercourse (World Health Organization [WHO], 2023). Infertility is estimated to impact approximately one in six people at some stage of their life (WHO, 2023). While no prevalence data exists denoting infertility rates in Muslim communities globally, the World Health Organization’s prevalence estimates do suggest that rates of infertility may be lower in Muslim-majority countries throughout the Middle East and North African region, though this data is not conclusive (WHO, 2023).

Culturally, however, experiencing infertility may present additional pressures for Muslim couples, and the condition can be highly stigmatized in Muslim communities (Reaves & Hauck, 2019). The impacts of the cultural and religious pressures to have children, combined with religious teachings that infertility is a ‘decree from Allah’ (Butt & Shah, 2019), can mean that infertility is experienced as shameful. In fact, because parenthood is considered so central within Islam, and infertility is a direct barrier to this goal, one *hadith* (sayings and deeds of the Prophet Mohammed) recommends that Muslims avoid marrying barren women (Sunan Abi Dawud, 2050). These cultural and religious norms with regards to infertility have significant social and psychological costs for couples.

### Assisted Reproductive Technologies

Assisted Reproductive Technologies (ARTs) are fertility related treatments in which eggs, embryos, and sperm are manipulated with the goal of achieving pregnancy (Jain & Singh, 2023). The first ART-assisted human was born in 1978 (Steptoe & Edwards, 1978), and since then, countless advances in ART procedures and treatments have been made to improve outcomes for patients (Kamel, 2013). In the modern era, ARTs comprise a number of procedures and techniques including: Intrauterine insemination (IUI), a type of ART in which the sperms are placed in the uterine cavity at the time of ovulation; in vitro fertilization (IVF), where one or more fertilized eggs (embryos) are placed inside the uterine cavity with the hopes of attachment; gamete intrafallopian transfer (GIFT), a procedure similar to IVF though uncommonly used nowadays; zygote intrafallopian transfer (ZIFT), where an embryo developed in vitro is transferred laparoscopically into to the fallopian tube; and intracytoplasmic sperm injection (ICSI), a technique in which a single spermatozoon is injected directly into the egg prior to IVF insertion (Amjad & Rehman, 2021).

Just like with natural conception, successful pregnancy via ARTs require three things: fertile spermatozoa (sperm), fertile eggs, and a female reproductive system that is capable of carrying a pregnancy. Obtaining these three things can, in some cases, be difficult. Individuals with female reproductive systems are required to go through a series of hormone treatments to encourage oocyte maturation, after which the mature ovum are retrieved via a surgical procedure (Pellicer & Gomez, 2020). Men, on the other hand, provide the sample utilized in embryo creation through masturbation to achieve ejaculation. Lastly, to achieve pregnancy via ART requires a female reproductive system, usually the mother. However, as some cases of infertility may be related to a person’s inability to carry a pregnancy to full term, some couples may seek the assistance of a surrogate.

### ARTs and Islam

The use of assisted reproductive technologies in Muslim communities can be a contentious topic. Religiously, perspectives can differ depending on Islamic sect, school of thought, and individual interpretation. When it comes to the two largest Islamic sects—Sunni and Shi’a—divergences in religious jurisprudential rulings have led to vastly different understandings of the acceptability of certain ARTs in the respective communities (Khan & Konje, 2018; Serour, 2013).

This is particularly clear when it comes to third-party donations of gametes. In Shi’a-majority Iran, Ayatollah Ali Khamene’i issued a fatwa permitting the involvement of a third-party in couples’ fertility treatments, including through donations of eggs and sperm, as well as surrogacy (Serour, 2013). This fatwa made the acceptability of ART more flexible in Shi’a communities. However, in recent years, there has been some concern regarding the acceptability of using donated sperm. Consequently, many Shi’a scholars today forbid sperm donation (Serour, 2008). In comparison, Sunni perspectives are fairly unanimous on the prohibition on the use of any third-party material for artificial reproduction, and especially the use of donated sperm (Al-Bar & Chamsi-Pasha, 2015; Khan & Konje, 2018).

Despite these convergences on the use of third-party donations, both Sunni and Shi’a perspectives encourage the treatment of infertility through various ARTs (Serour, 2008). Modern fertility treatments are generally viewed as positive as they are the final way to facilitate procreation and parenthood, especially as adoption is often considered impermissible in Islam (Chaudhry, 2010). However, the permissibility of the use of ART is also contingent on the relationship status of the patient; ART is exclusively restricted to married, heterosexual couples. Single women or homosexual couples are not considered religiously permitted to receive assisted reproduction (Shamani et al., 2007).

It is important to note that religious rulings on certain practices pertain to how the religious establishment views a particular issue, and not necessarily to how that issue is interpreted or practiced within Muslim communities. There is a wide variety of personal perspectives on ARTs’ alignment with Islamic principles, some of which will be discussed in this study.

## Research Methods

### Overview

This research seeks to answer the following research question:

*What are the barriers impacting Muslim individuals and couples' access to equitable Assisted Reproductive Technology (ART), with particular, though not exclusive focus on: religious belief and interpretation; community and familial norms; and cultural/religious capacity of service providers?*

To explore the research question, a scoping review was undertaken. The review follows the framework set out by Arksey and O’Malley (2005) and the additional recommendations of Levac et al. (2010). The reporting of the findings follows the Preferred Reporting Items for Systematic Reviews and Meta-Analysis extension for Scoping Reviews—PRISMA-ScR (Tricco et al., 2018).

### Search Strategy

A four-step search strategy identified relevant studies. First, an initial limited search using Google Scholar and Medline (OVID) was undertaken, followed by a review of words used in titles, key words, subject headings, and index terms to identify relevant search terms (Box 1). A second search using the identified search terms was undertaken using MEDLINE, PsycINFO and 100 + other databases via OVID. No lower time limit was imposed as ART has only become widely available in the past 2–3 decades. Two researchers reviewed and discussed the results. Finally, a hand-search was undertaken using the reference lists of relevant literature to identify studies that may not have been picked up through the original searches. This review includes studies up until December 2023. No new articles beyond this date are included (Table 1).

**Table 1:** Search terms used in databases

*Search terms used in search of OVID databases for the first search of the intersection between harm or violence toward animals and DA:*	
1	(IVF or ART or infertil* or fertil* or in$vitro fertili?ation or assisted reproductive treatment$ or assisted reproductive technolog* or fertility treatment$ or egg retrieval$ or insemination or assisted reproduction or artificial insemination or semen analys*s)
2	(Islam* or Muslim$ or Shar*a)
3	1 and 2
4	limit 3 to yr = ”- Current”

### Study Selection

Studies were extracted from the academic research databases Medline (Ovid), SCOPUS, and Web of Science, then uploaded into Covidence online screening tool. Duplicate articles were identified by Covidence and removed, with additional duplicates removed manually by the researchers. In the initial screening phase, both researchers conducted double-blind review of the titles and abstracts—two negatives would mark the article as irrelevant, two positives as relevant, and two different votes (yes, no, or maybe) would flag the article as a conflict. Conflicts were resolved by both reviewers after discussing the article together. Following this, the full text of the articles were reviewed following the same protocol. Articles that were screened as relevant were moved on to the data extraction phase, where both researchers reviewed and extracted the data for analysis.

### Selection Criteria

Initially, the criteria for screening in studies for review was kept broad to get a full understanding of the scope of research into the topic. Studies were screened in if they focused on the intersection between Islam and/or Muslim communities and assisted reproductive technologies and/or fertility. Following further analysis, only studies that had human participants were included, and further to that, only those which focused on Muslim communities’ experiences of ART, community and patient beliefs and attitudes toward ART, or fertility sector professionals’ views surrounding access barriers for Muslim couples and communities. As much of the literature was analysis of the legal contexts surrounding ART in Muslim-majority countries, or the theological arguments for and against certain procedures, this left only a small number of studies relative to the initial search. Some studies also spoke of barriers unrelated to intracommunity religious and cultural issues (e.g., financial barriers to ART access, language barriers to ART access, racism in ART treatment). Though important, this is not the focus of this review.

## Results

**Fig. 1:**
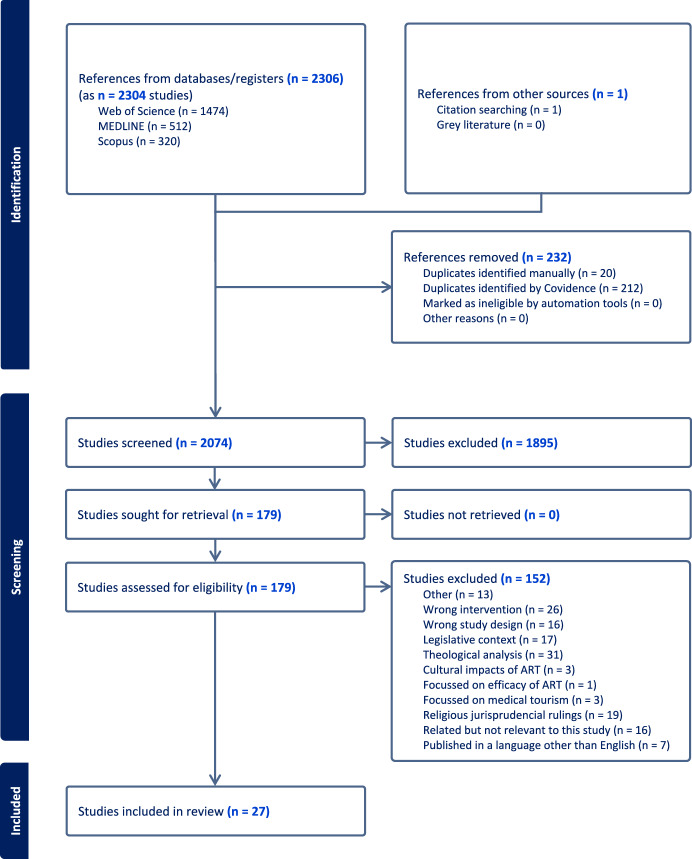
Studies identified through search

A total of 2306 studies were extracted from the academic research databases Medline (Ovid), SCOPUS, and Web of Science, then uploaded into Covidence online screening tool. Of these, 212 were automatically removed and 20 were manually identified as duplicates and removed. This left a total of 2073 studies to be screened based on title and abstract. Of these, 1895 did not fit the inclusion criteria, leaving 178 for a full-text analysis. From this analysis, 26 articles were found to be relevant for this study (see Fig. 1 below). An additional study was found during the hand search, bringing the total number of included studies to 27. No additional gray literature studies were found. A list of articles and their details included in this study can be found in Appendix 1.

### Overview of Studies

There was a range of methodologies and foci of the studies that explored Muslim communities’ views on and experiences with ART. 10 studies were qualitative, utilizing data from interviews and/or focus group discussions with patients experiencing infertility (Batool & de Visser, 2016; Bokek-Cohen et al., 2022a, 2022b; Gameiro et al., 2019; Husain & Imran, 2020; Martin-Anatias & Davies, 2023; Scully et al., 2017), both patients and medical practitioners (Behjati Ardakani et al., 2022; Bokek-Cohen et al., 2021; Murad et al., 2014), and in one study, patients, physicians, surrogates, egg donors, and religious leaders were interviewed (Hörbst, 2016).

7 studies were cross-sectional, focusing on quantitative data at one point in time (Ahmadi & Bamdad, 2017; Aslan et al., 2017; Bokek-Cohen & Tarabeih, 2022; Bokek-Cohen et al., 2022a, 2022b; Iliyasu et al., 2013; Isikoglu et al., 2006; Salehi et al., 2015). Data for these studies were collected through surveys to assess the target cohorts’ views on and attitudes toward ART.

Another 7 studies were ethnographies (Blell, 2018; Clarke & Inhorn, 2011; Inhorn, 2004, 2006a, 2006b, 2007; Monroe, 2022). In these studies, data were collected over long periods in the form of interviews with patients and practitioners, field notes, and observational methods to assess views on and experiences with accessing or providing ART.

Two studies (Igbolekwu et al., 2022; Lestari et al., 2022) used a mixed methods approach; Igbolekwu et al.’s (2022) research collected quantitative data through questionnaires with Muslim and Christians, and qualitative data through interviews with cultural/religious leaders to assess these communities’ views/knowledge on artificial insemination. Lestari et al.’s (2022) study also used questionnaires and interviews, though focused on students and faculty members of a university to measure beliefs and acceptability around the use of IVF.

The final article was a literature review (Reaves & Hauk, 2019). This review sought to collate findings related to Muslim migrant and refugee experiences of infertility in Middle Eastern countries. It included articles that focused on the use of ART among Muslim refugees, the impact of infertility on this cohort, and barriers to care.

The studies were based in a range of countries, though primarily Muslim-majority countries. Only 5 were based in Muslim-minority countries (Blell, 2018; Bokek-Cohen et al., 2022a, 2022b; Gameiro et al., 2019; Martin-Anatias & Davies, 2023; Scully et al., 2017). Two were comparisons of experiences of ART or infertility in Muslim-majority and Muslim-minority countries, with one study based both in the UK and Pakistan (Batool & de Visser, 2016), and another based in Mali and Uganda (Hörbst, 2016). Further descriptions of the studies included in this research can be found in Appendix 1.

#### Theme 1

Socio-cultural factors impacting patients’ views and acceptance of ART

There were many socio-cultural factors present within the studies that influenced use of and access to ART among the populations studied. The social pressure to have children was a consistent theme observed within many of the studies (Batool & de Visser, 2016; Blell, 2018; Bokek-Cohen et al., 2021, 2022a, 2022b; Gameiro et al., 2019; Martin-Anatias & Davies, 2023; Reaves & Hauck, 2019). These pressures left patients feeling stigmatized by their conditions (Batool & de Visser, 2016; Blell, 2018; Inhorn, 2004), and this social stigma presented as a barrier to accessing ARTs (Husain & Imran, 2020). For Muslim patients who did decide to access fertility treatments including ART, secrecy was sometimes adopted to maintain privacy, especially in contexts where patients were accessing treatments which were considered religiously impermissible by their respective communities (Bokek-Cohen et al., 2021, 2022a, 2022b; Inhorn, 2004; Reaves & Hauck, 2019).

The preference and desire for biological children was present across many of the articles included in this study (Behjati Ardakani et al., 2022; Clarke & Inhorn, 2011; Inhorn, 2006a; Martin-Anatias & Davies, 2023). This preference presented as a barrier to accessing ART as it meant that some patients delayed or refused certain procedures, particularly the use of third-party gametes (Behjati Ardakani et al., 2022; Blell, 2018; Clarke & Inhorn, 2011; Inhorn, 2004). Yet some studies showed that parents who were hesitant to accept the use of donated gametes, nevertheless preferred non-biological children over no children (Behjati Ardakani et al., 2022; Martin-Anatias & Davies, 2023). Other studies, however, showed that couples often chose childlessness over violating religious prohibitions on donated gametes (Inhorn, 2006a, 2006b).

Some studies also suggested that attitudes and barriers toward ARTs for infertile Muslim couples can differ depending on the country and location of treatment (Batool & de Visser, 2016; Inhorn, 2004). One study by Batool and de Visser (2016), for instance, compared Muslim women’s experiences of infertility in the United Kingdom and Pakistan. While there were some similarities in terms of participants’ desires for motherhood, women in Pakistan faced more societal and familiar pressure for children, though also had more religious reservations about IVF and the use of donated gametes (Batool & de Visser, 2016). Inhorn’s (2004) research compared men’s experiences of infertility in Cairo and Beirut. While in Cairo, men were generally more secretive regarding their infertility due to social stigma, in Beirut, infertility was more accepted as a medical problem (Inhorn, 2004). This meant that there were fewer social barriers to accessing ART in Lebanon than in Egypt (Inhorn, 2004). Another study (Inhorn, 2006b) focused on experiences of accessing donor gametes in Iran and Lebanon, and highlighted a case study of a participant who had also received treatment in the United States. This participant spoke about the differences in attitudes toward egg donation in the US compared to Lebanon, and specifically that negative attitudes made it difficult to access the required treatment in the latter country (Inhorn, 2006b).

#### Theme 2

Religious and legal barriers to treatment

The issue of legal and religious barriers to accessing or accepting ARTs was consistent across many of the studies included in this review (Batool & de Visser, 2016; Behjati Ardakani et al., 2022; Bokek-Cohen et al., 2021, 2022a, 2022b; Inhorn, 2006b; Lestari et al., 2022). In several cases, due to the setting of the study, the religious and legal barriers were connected, as the legal system of certain countries were tied in with *shari’a* and *fiqh* (Behjati Ardakani et al., 2022; Bokek-Cohen et al., 2022a, 2022b; Monroe, 2022).

Patients accessing treatments overcame these barriers through various means, though there remained differing views among participants across and even within studies surrounding which aspects of ART were religiously, legally, and even personally accepted. Studies showed that Muslim couples wished to conceive in a religiously permitted way (Martin-Anatias & Davies, 2023; Monroe, 2022; Murad et al., 2014), whatever that meant to them. Consistently, the most contentious aspect of ART was the use of third-party donated gametes (Aslan & Elter; 2017; Batool & de Visser, 2016; Behjati Ardakani et al., 2022; Hörbst, 2016; Inhorn, 2006a, 2006b; Martin-Anatias & Davies, 2023). Husbands/men in particular displayed aversions toward the use of donated sperm (Behjati Ardakani et al., 2022; Hörbst, 2016; Inhorn, 2006b), though this was a concern for women too (Batool & de Visser, 2016; Martin-Anatias & Davies, 2023). These aversions were connected to religious and legal permissibility of using donated gametes (Inhorn, 2004, 2006b) though in some cases, the rationale behind the disapproval or refusal of this service was unclear or unstated in the studies (Behjati Ardakani et al., 2022).

Another contentious religious issue with regards to ART was related to the collection of semen samples for IVF (Inhorn, 2007; Murad et al., 2014). One study by Inhorn (2007) looked at Lebanese and Egyptian Muslim men’s experiences providing semen samples, with specific focus on religious guilt and shame related to masturbation. Murad et al.’s (2014) study likewise showed that some patients accessing ARTs have questions surrounding the religious implications for engaging in masturbation to provide a semen sample. Although this was a theme that was absent from the rest of the studies included, it is important to highlight as Islam’s religious position on masturbation—that it is prohibited—presents a significant barrier to ARTs. In the case of Inhorn’s (2007) study, the experience of providing semen samples raised feelings of shame, sin, and impurity.

Across the studies, patients adopted different strategies to manage or overcome religious and legal barriers to accessing ARTs. Some studies even included examples of patients engaging in illegal or subversive activity to overcome legal prohibitions on certain procedures—for instance asking doctors to use donated sperm without the wife’s consent or knowledge (Behjati Ardakani et al., 2022; Inhorn, 2006a), asking the doctor to use donated sperm without the husband’s knowledge (Bokek-Cohen et al., 2021), or accessing ‘underground’ markets of third-party donated gametes (Bokek-Cohen et al., ).

Many studies reported that patients access religious guidance and advice from clerics (Behjati Ardakani et al., 2022; Bokek-Cohen et al., 2021, 2022a, 2022b; Clarke & Inhorn, 2011; Martin-Anatias & Davies, 2023). Sometimes this guidance is accepted and influences couples’ acceptance of certain procedures (Clarke & Inhorn, 2011). In other cases, patients may change clerics until they can find someone who is willing to religiously endorse their preferred treatment (Behjati Ardakani et al., 2022; Martin-Anatias & Davies, 2023), or come to their own decision on the matter, contrary to the advice of their faith leaders (Bokek-Cohen et al., Inhorn, 2006b; Martin-Anatias & Davies, 2023). On the other hand, one study by Hörbst (2016) demonstrated that some Muslim couples do not seek advice from religious clerics surrounding ART, and instead believe that questions related to the religious ethics of ART are a private negotiation with God.

#### Theme 3

Social and community attitudes surrounding ART

In the surrounding context of patients across the studies who were accessing ARTs were the perceptions of fertility treatments in their respective communities. Our review analyzed several studies that focused solely on community perceptions to demonstrate how Muslim communities, on a wider level, view and accept the use of ART and certain types of procedures that fall under ART. These community perceptions were important to understand as they inevitably impact choices decision-making surrounding ART within the societies in which the studies are performed.

The studies that focused on community perceptions of ARTs were performed in Iran (Ahmadi & Bamdad, 2017), Turkey (Aslan & Elter, 2017; Isikoglu et al., 2006), Israel (Bokek-Cohen & Tarabeih, 2022; Bokek-Cohen et al., 2022a, 2022b), Nigeria (Igbolekwu et al., 2022; Iliyasu et al., 2013), and Indonesia (Lestari et al., 2022). While there were variations across these studies in terms of the levels of permissibility of certain ARTs, attitudes toward the use of donated eggs and sperm were consistently the most negative (Ahmadi & Bamdad, 2017; Aslan & Elter; 2017; Bokek-Cohen et al., 2022a, 2022b; Bokek-Cohen & Tarabeih, 2022). It should also be mentioned that in studies where participants were asked about this factor, marriage was a clear condition of ARTs’ permissibility (Lestari et al., 2022).

Interestingly, in some studies where both men and women participated, the opposition to the use of donated materials was also gendered; women were less likely to disapprove of using donated eggs, embryos, or sperm than men (Ahmadi & Bamdad, 2017; Bokek-Cohen & Tarabeih (2022). However, in a separate study, the opposite was true—men displayed higher approval (Isikoglu et al., 2006), and in another still, results turned up no significant gender difference in levels of acceptance of different types of ART (Bokek-Cohen & Tarabeih, 2022). Among the cross-sectional studies focusing on levels of acceptance of ARTs in various populations (Ahmadi & Bamdad, 2017; Aslan & Elter; 2017; Bokek-Cohen et al., 2022a, 2022b; Bokek-Cohen & Tarabeih, 2022; Iliyasu et al., 2013; Isikoglu et al., 2006), there were disparities in terms of the variables which correlated with higher or lower acceptance of ARTs. In Bokek-Cohen and Tarabeih’s (2022) study, for example, levels of religiosity were not associated with acceptance of any form of ART, while in Bokek-Cohen et al.'s, (2022a) study, a higher level of religious observance was significantly (p < 0.01) associated with objection toward egg, embryo, and sperm donation. In Ahmadi and Bamdad’s (2017) study, the variations in levels of approval were not impacted by age, education, or marital status, but were significantly impacted by employment status (p < 0.05), and in Igbolekwu et al.’s (2022) study, Sunni Muslims were more likely to be accepting of artificial insemination than Shi’a Muslims. Evidently, correlative variables differed across the studies, and as no study investigated moderating factors, it is difficult to assess what the significant associations signify, where they did exist.

It is also worth noting that the populations included in each study differed. Ahmadi and Bambad’s (2017) study included male and female community members in Iran; Aslan and Elter’s (2017) study included fertile and infertile women in Turkey; Bokek-Cohen and Tarabeih’s (2022) study included married Sunni Muslims in Israel; Bokek-Cohen et al.’s (2022a, 2022b) study included Sunni Muslim Palestinian physicians and medical students; Igbolekwu et al.’s (2022) study included Muslim and Christian community members and religious leaders in Nigeria; Iliyasu et al.’s (2013) study included married Muslim couples in Nigeria; Isikoglu et al.’s (2006) study included Muslim residents of two undisclosed cities in Turkey; and Lestari et al.’s (2022) study included university students in Indonesia.

#### Theme 4

Nature and quality of care

Several studies reported on patients’ experiences with relation to the quality of treatment, support from practitioners and professionals, and culturally competent care they had received (Blell, 2018; Gameiro et al., 2019; Scully et al., 2017). Other studies focused on practitioners’ perceptions of ARTs (Bokek-Cohen et al., ).

While experiences and quality of care differed widely across studies, the issues that arose were also connected to the location and population of the studies. For example, studies set in western contexts were more likely to focus on low levels of practitioner cultural, religious, and linguistic capacity, and structural barriers which impact patient experiences and quality of care (Blell, 2018; Gameiro et al., 2019; Martin-Anatias & Davies, 2023; Scully et al., 2017). In comparison, studies set in Muslim-majority countries highlighted issues such as costs of treatment, legal barriers, and differences of religious understanding between patients and practitioners (Husain & Imran, 2020; Inhorn, 2004, 2006b).

One study that focused on Pakistani Muslim men’s experiences accessing fertility treatment in the UK, for instance, found that staff from Muslim backgrounds were seen as having higher cultural knowledge when treating Pakistani patients (Blell, 2018). This was viewed as important as Pakistani men had high levels of disengagement from treatment, characterized by a refusal to provide semen samples and failure to attend appointments. In addition, language support was infrequently offered, leading to confusion and miscommunication surrounding what the various ART treatments involved (Blell, 2018). Similarly, Gameiro et al.’s (2019) study, set in Wales, showed that Muslim women receiving fertility treatments faced cultural, religious, and linguistic barriers which can intensify the already high levels of stress related to accessing treatment. Martin-Anatias and Davies’ (2023) study, set in New Zealand, highlighted practitioners’ insensitivity to patients’ religious beliefs surrounding which aspects of ART they would and would not accept as part of their treatment, and Scully et al.’s (2017) study, set in the UK, showed that Muslim patients felt that healthcare professionals were insensitive or disinterested in their religious considerations. Consistently across these studies based in western countries, many patients from Muslim backgrounds faced difficulties receiving culturally competent care from practitioners. Consequently, participants often felt compelled to do their own research and seek external advice from non-medical sources to inform their care choices.

It is also interesting to note the results from Bokek-Cohen et al.’s (2022a, 2022b) study, which investigated Palestinian Sunni Muslims’ perceptions of ARTs. The results of this study suggested that among the cohort included, the vast majority of clinicians, regardless of training level, age, and religious observance, adopt positions on ARTs which are largely in line with Islamic rulings on the topic. That is; that donated gametes, and particularly donated sperm, is not permissible (Bokek-Cohen et al., 2022a). Although the results of this study cannot be extrapolated to Muslim clinicians in general, it does indicate that there are Muslim clinicians who have personal beliefs surround ARTs that may or may not impact the quality and nature of fertility care. This is supported by Monroe’s (2022) study, set in Qatar, which showed that although third-party donation was illegal in Qatar, physicians still told patients that these options existed elsewhere. Interestingly—and conversely to the above—there were also studies included in this review which demonstrated that some Sunni Muslim clinicians, despite the relatively consistent belief among Sunni clerics that third-part gamete donation is prohibited in Islam, perform IVF procedures with donated materials (Bokek-Cohen et al., 2021, 2022a, 2022b; Hörbst, 2016). Even in cases where the physicians themselves believe the procedures to be religiously prohibited, they may still inform patients of these treatment options to allow them to make their own informed decisions (Hörbst, 2016; Monroe, 2022).

However, accessing ARTs in Muslim-majority countries did not always guarantee a high level of religiously and culturally competent care. Inhorn’s (2007) study, which focused on Muslim men’s religious reservations related to providing semen samples via masturbation, highlighted how little clinics may be doing to assuage male patients of their reservations surrounding this necessary aspect of ART. In fact, Inhorn (2007) argues that clinics may be exacerbating men’s anxieties when privacy cannot be guaranteed, or when pornography is made available—the use of which compounds feelings of shame (Inhorn, 2007). Similarly, Murad et al.’s (2014) study, set in Malaysia, showed that both patients and clinicians have questions regarding what is and is not religiously permissible when it comes to receiving and providing ART. Consequently, patients have anxieties about accepting treatment that is not *shari’ah* compliant (Murad et al., 2014).

#### Theme 5

Gender, masculinity, and ARTs

Several studies explored how gender and masculinity could impact Muslim men and women’s experiences of ART treatments (Batool & de Visser, 2016; Blell, 2018; Bokek-Cohen et al., 2021; Inhorn, 2004).

Although infertility was often a shared issue between the couples, women were seen to be driving the active pursuit of fertility treatments and ART, with men sometimes taking a more passive role in treatment (Batool & de Visser, 2016). This often led to frustration from women, particularly when couples were experiencing male-factor infertility. Men’s more resistant or avoidance responses was indicative of the cultural perception that infertility is a woman’s issue, and several studies did mention the disproportionate blame that is often placed on women in cases of infertility (Batool & de Visser, 2016; Blell, 2018; Gameiro et al., 2019; Lestari et al., 2022; Reaves & Hauck, 2019).

Going further, in some studies, the belief that infertility was a woman’s issue led to male patients refusing to engage in necessary treatments (Blell, 2018; Inhorn, 2007). Cultural expectations, feelings of shame and stigma around infertility, and being unwilling to engage in necessary aspects of treatment (such as the provision of a semen sample for IVF) intersected with men’s views of their own masculinity (Blell, 2018; Inhorn, 2007). Some men reported feeling shame, inferiority, and threats to masculinity as a result of their infertility (Bokek-Cohen et al., 2021; Inhorn, 2004; Reaves & Hauck, 2019). And while these feelings caused some men to withdraw from accessing ARTs (Blell, 2018) in other cases, the stress and pressure of being unable to conceive led some men to access aspects of ART treatments that were religiously prohibited in their communities (Bokek-Cohen et al., 2021; Inhorn, 2007).

## Discussion

Family and procreation are of central importance both culturally and religiously in Muslim communities. This can mean that when infertility is experienced by couples, there are added pressures to find effective treatment, such as ART. Considering Muslim communities’ specific cultural, religious, and personal positions when it comes to accessing infertility treatments, this research sought to identify what barriers impact Muslim individuals and couples’ access to equitable ART treatments, as evidenced in the international literature. We aimed to focus primarily, though not exclusively on three areas: religious belief and interpretation; community and familial norms; and cultural/religious capacity of service providers.

Our results showed that Muslim communities face several primary barriers and considerations when accessing ART. Foremost, the cultural pressure to have children often drives couples to seek out treatment in the first instance. Yet this cultural pressure also impacts the type of ART that patients are open to accepting. 

Community perceptions regarding certain types of ART—driven by underlying religious beliefs and teachings—render treatment options including third-party donation undesirable for many patients (Behjati Ardakani et al., 2022; Batool & de Visser, 2016; Aslan & Elter; 2017; Hörbst, 2016; Inhorn, 2006a, 2006b; Martin-Anatias & Davies, 2023). Overall, couples highly valued religious prescriptions on ART, and wished to conceive in what they considered a ‘religiously permitted’ way (Martin-Anatias & Davies, 2023; Monroe, 2022; Murad et al., 2014). At the same time, several studies also showed that there are couples who, despite religious teachings that prohibit certain types of ARTs, choose to come to their own decision surrounding the acceptability of treatments (Bokek-Cohen et al., 2021, 2022a, 2022b; Inhorn, 2006b; Martin-Anatias & Davies, 2023). This demonstrates that establishment positions on ART—which existing research shows often prohibit the use of third-party donations and especially or specifically donated sperm (Khan & Konje, 2018; Serour, 2013)—do not always align with how patients behave in practice. While religious teachings remain a barrier to patients, especially where they are unable to access certain types of treatments due to religiously based legal prohibitions (Behjati Ardakani et al., 2022; Monroe, 2022), they do not necessarily mean that all couples will follow the religious guidance they receive.

In the context surrounding patients’ access to and acceptance of ARTs were the community perceptions of these treatment options. Our results showed that consistently across the countries included in the cross-sectional studies of community attitudes toward ARTs, the use of donated eggs and sperm were viewed as the most negative (Ahmadi & Bamdad, 2017; Aslan & Elter; 2017; Bokek-Cohen & Tarabeih, 2022; Bokek-Cohen et al., 2022a, 2022b). With this context in mind, it becomes clearer why patients often adopt secrecy when going through treatment (Bokek-Cohen et al., 2021, 2022a, 2022b; Inhorn, 2004; Reaves & Hauck, 2019). The fear and shame of judgment both for the initial infertility as well as for the use of “impermissible” treatment options is likely to impact patients’ mental health and wellbeing throughout their infertility journey. Research shows that experiencing infertility and having to go through treatment can have many negative mental health implications, some of which can continue well after treatment has discontinued (Gameiro et al., 2016). At the same time, social support has been shown to be a protective factor for mental health outcomes among numerous populations across the globe, and can also influence health behaviors such as continuation with treatments (Umberson & Montez, 2010). Perceiving adequate support from social networks during IVF specifically is correlated with more positive mental health outcomes during treatment (Gameiro et al., 2016). There is therefore a potential for increased risk of negative mental health and wellbeing among couples who feel compelled to maintain secrecy, and who fear judgment surrounding disclosure of the diagnosis as well as the treatment received.

When looking at the quality-of-care Muslim couples receive when accessing ART, the results across studies showed a variety of experiences. Looking comparatively between studies set in Muslim-minority (namely western) and Muslim-majority contexts, results demonstrated that the primary barriers for Muslim patients in the former contexts were low levels of practitioner cultural, religious, and linguistic capacity, and structural barriers which impact patient experiences and quality of care (Blell, 2018; Gameiro et al., 2019; Martin-Anatias & Davies, 2023; Scully et al., 2017). In the latter, barriers included costs of treatment, legal barriers, and differences of religious understanding between patients and practitioners (Husain & Imran, 2020; Inhorn, 2004, 2006b).

Interestingly, though not the most central theme across studies, gendered aspects of care were highlighted both explicitly and implicitly in the research analyzed. While women often felt the most pressure and blame for the infertility (Batool & de Visser, 2016; Blell, 2018; Gameiro et al., 2019; Lestari et al., 2022; Reaves & Hauck, 2019), men were likewise navigating gendered pressures and expectations. Some men reported feeling shame, inferiority, and threats to masculinity due to their infertility (Bokek-Cohen et al., 2021; Inhorn, 2004; Reaves & Hauck, 2019). Men’s fears surrounding their masculinity resulted in various responses; some men became avoidant, refusing to engage in necessary treatments (Blell, 2018; Inhorn, 2007), while others moved to accept certain types of treatment they would otherwise have rejected (Bokek-Cohen et al., 2021; Inhorn, 2007). While issues of masculinity can certainly damage ART prospects and options, they may also be a motivating factor.

It was particularly notable when analyzing the studies included in this review that the vast majority of the research was conducted in Muslim-majority countries, or countries with large Muslim minorities. There is only a small amount of literature that focuses on how Muslim communities in the west experience infertility and the barriers to accessing ARTs. This gap in the literature is important to highlight, as Muslim communities in the west—and especially migrant and refugee Muslims—face many compounding issues which are likely to negatively impact their access to appropriate fertility treatments. For example, in Australia, where the authors of this research are based, Muslim communities are overrepresented in lower-income categories and significantly underrepresented in higher income categories (Hassan, 2018), may experience racism and Islamophobia in the healthcare setting (Ben et al., 2023), and often face language barriers and a lack of quality interpretative support when accessing Australian systems and services (Casimiro et al., 2007; Henderson & Kendall, 2011). On top of this, migrant or refugee Muslims may be on visa sub-classes which prohibit their access to Australia’s subsidized or free healthcare (Ziersche et al., 2020). These are all issues which have the potential to drastically impact Muslim communities’ access to ART. Further, it is likely that in the west, Muslim patients will experience the same levels of religious trepidation and questions with relation to their ART treatment options as in Muslim-majority countries, though will be doing so without the support of a practitioner who has knowledge and understanding of these issues and considerations. At the same time, it may be the case that other barriers are minimized in western contexts. Without increased research into Muslim communities’ experiences of accessing ART in the west, it is difficult to know definitively.

These findings have implications for policy and practice among healthcare practitioners offering ART treatment options to Muslim patients. Firstly, Muslim patients accessing ART place a high value on the religious contexts surrounding their care. The provision of culturally and religiously appropriate care for Muslim patients—whether in the Muslim-majority or Muslim-minority context—is therefore paramount. Research demonstrates that improved health and social care outcomes can be achieved for Muslim patients when health interventions are religiously tailored to their personal needs, and when notions of health are extended to include cultural and spiritual domains important to the patient (McLaren et al., 2022). It is therefore integral that practitioners are mindful of this context, and that they support their patients in a culturally—and religiously—safe manner to come to their own conclusions regarding their acceptance of treatment options. At the same time, where options have been exhausted, it should be ensured that patients have been provided with adequate mental health support.

## Limitations

There are some limitations to this study that should be addressed. Firstly, it is important to highlight that although this review has primarily included studies based in countries where English is not the primary language, we have only included research published in English. There is therefore likely to be several studies conducted in languages other than English that are not analyzed here, but which have findings relevant to the research question. Furthermore, due to the diverse views and beliefs with regards to Islam and ART, there is a wide range of heterogeneity of the research included in this study. We therefore advise caution when attempting to generalize results to a specific target population.

## Conclusion

This review makes an important contribution toward understanding the nuances, barriers, and struggles of infertile Muslim couples and their reproductive choices regarding ARTs. Despite moral and religious challenges, many Muslims around the world seek to find ways to achieve their desire of having children. Children are viewed as a religious investment and hold great importance in all cultures that have embraced Islam. Consequently, the mechanisms and means that allow Muslim couples and individuals to realize this goal should be analyzed and understood to facilitate the best possible care and treatment options for Muslim patients. Our review collates and analyzes the research to encourage its use within policy and practice in healthcare settings. Further research, based in western countries, should be conducted to better understand how these contexts can support Muslim patients accessing ART.

## Appendix 1

## Studies Included in Analysis

**Table:** 

References	Study Design	Study Population	Research Methods	Main Findings
Ahmadi, A., & Bamdad, S. (2017). Assisted reproductive technologies and the Iranian community attitude towards infertility. *Human Fertility,* 20(3), 204–211. 10.1080/14647273.2017.1285057	Cross-sectional study	405 residents of Shiraz, Iran (276 women and 129 men)	Participants were selected through cluster sampling to complete a two-part questionnaire that included questions related to demographics as well as attitudes towards and adoption of fertility treatments. Factor analysis was used to validate the reliability of the questionnaire. Significance testing of the data was undertaken to explore associations between explanatory variables	The results indicated that respondents did not support all types of assisted reproduction. Amongst modern infertility treatment methods, IVF (using husband’s sperm and wife’s egg) was the most widely accepted—76% of women and 78.4% of men endorsed IVF as an acceptable solution. Gestational surrogacy and the use of donated gametes were less accepted. Participants also largely disagreed with the use of donated sperm. Demographic variables including gender, marital status, age, education and employment status were linked to significant differences in public opinion
Aslan, M.M., Ugurel, V., & Elter, K. (2017). The attitudes of fertile and infertile women to Oocyte donation in a Muslim and Secular population. *Pakistan Journal of Medical Science*, 33(5),1260–1264. 10.12669/pjms.335.13556	Cross-sectional study	The participants consisted of fertile women (n = 133) who had at least one healthy living child spontaneously conceived without any fertility treatment and infertile women (n = 133) who were diagnosed with primary infertility. Ages 18 to 45 years, living in Turkiye	Participants were provided with a questionnaire that gathered demographic information as well as information about participants' knowledge regarding, views on, and acceptance of oocyte donation. They were also asked about their husband/partners' views on oocyte donation. Statistical analysis was performed to identify associations between variables	88% of fertile women and 82% of infertile women answered negatively about willingness to have children by oocyte donation. Similarly, most of the women in both groups declared negative answers about their husbands’ attitudes to oocyte donation. Participants were also against donating their own oocytes, with 91% of fertile women and 81% of infertile women stating that they would not do so. Infertile women (91.0%) compared to fertile women (78.2%) had a more positive attitude to the issue of “oocyte acceptance from close relatives.”
Batool, S.S., & de Visser, R.O. (2016). Experiences of infertility in British and Pakistani women: A cross-cultural qualitative analysis. *Health Care for Women International*, 37(2), 180–196. 10.1080/07399332.2014.980890	Qualitative Study	14 involuntarily childless women who has been in a relationship for more than two years and were pursuing fertility treatment. Eight women were living in the UK, six women were living in Pakistan	Participants were recruited via a gynecologist and snowball sampling. In-depth interviews were conducted, during which demographic details were collected, and participants were asked about their desire for motherhood, experiences of infertility diagnosis and treatment, impacts of infertility, and coping responses. Data was coded and analyzed through an idiographic case-study approach	Participants had a strong desire for motherhood, and for the Pakistani participants, motherhood was further valued due to the status it gave them in the eyes of members of their extended family. Women in Pakistan appeared to be more apprehensive. They were often unsure about the reason for infertility, and even if a clear diagnosis had been given, effective treatment was not always available. Amongst Pakistani participants, the diagnosis of infertility brought fears of marital breakdown due to the desire for patrilineal inheritance. Participants on the whole avoided disclosing their infertility to family and community members. Pakistani participants expressed social and religious reservations about IVF, which related to Islamic prohibitions on donor insemination
Behjati Ardakani, Z., Navabakhsh, M., Ranjbar, F., Mehdi Akhondi, M., & Mohseni Tabrizi, A. (2022). Step-by-step decision-making process in third party assisted reproduction: a qualitative study. *Human Fertility*, 25(3), 487–498. 10.1080/14647273.2020.1817579	Qualitative Study	20 infertile patients (men and women) including 3 couples and 14 individuals who were evaluated or treated for the use of third-party reproduction at one treatment center in Tehran, Iran. In addition, 12 specialists in the field of reproductive treatment, research, or religious knowledge were also recruited for interview from different infertility clinics throughout Tehran	A purposive sampling method selected participants who had accessed pregnancy/parenthood through the involvement of a third-party (i.e. oocyte, embryo, or sperm donation). Of the 20 participants who were patients, 15 had children from this treatment and 5 were pregnant. Semi-structured interviewed were conducted to gather an understanding of how participants had come to the decision to utilize third-party reproduction, and what their experienced had been. Content analysis was performed	The results showed that selection of the treatment options in infertile couples has occurred in a gradual and step-by-step process. Using donated materials was difficult to accept for many participants. However, participants preferred using donated gametes (sperm/oocytes) to childlessness due to infertility and divorce stigma. Religion was a significant barrier to accepting treatment. Some participants sought out advice from religious leaders, with mixed results—while some participants' Marja (Twelver Shia religious cleric) endorsed the use of third-party gamete donations, others did not. Participants also hid their treatment method from others to allay their concerns resulting from cultural taboos and prevent any damage to their identity in terms of fertility capability and to their child’s identity into social parents and being viewed as second class citizens
Blell, M. (2018). British Pakistani Muslim masculinity, (In)fertility, and the clinical encounter. *Medical Anthropology*, 37(2), 117–130. 10.1080/01459740.2017.1364736	Ethnography	Infertile British Pakistani Muslims and relevant health professionals living in North East England	Participant observation and recruitment took place in an IVF clinic in North East England. Six interviews were conducted with clinic staff, and 15 interviews were conducted with British Pakistani infertility patients (six couples and three individual women). Interviews aimed to gather an understanding of men's experiences of and the cultural context when accessing fertility treatments. Data was also gathered through observation and via other informants in the healthcare setting. Data was coded and analyzed based on principles of grounded theory	Staff from Muslim cultural or ethnic backgrounds were seen to have higher cultural knowledge when treating Pakistani patients. Male patients from Pakistani backgrounds often disengaged. This lack of engagement was characterized by a refusal to provide semen samples and failure to attend appointments with their wives after initial consultations. Adequate language support was infrequently offered, impacting communication about the ART procedures between patient and professional. Wider family networks had an impact on treatment experiences. Stigma, taboo, and fears surrounding how IVF would impact patrilineages were all a factor
Bokek-Cohen, Y., Gonen, L.D., & Tarabeih, M. (2022). The ethical standards of Sunni Muslim physicians regarding fertility technologies that are religiously forbidden. *Journal of Religion and Health*, 61(4), 2876–2904. 10.1007/s10943-022-01583-9	Cross-sectional study	689 Physicians or graduating medical students who were of Sunni Muslim faith. All participants are Palestinians living in Israel	This study was intended to examine to what extent Sunni Muslim physicians support the provision of egg, sperm and embryo donation; surrogacy and adoption; and gender selection. Snowball and convenience sampling was used to recruit participants. An online questionnaire was sent to eligible participants, comprised of vignettes depicting hypothetical scenarios of couples accessing ART. participants rated responses on a Likert scale. Significance testing was performed to identify associations between variables	Overall, the highest level of objection was towards sperm donation, followed by egg and embryo donation. Gender selection for a male foetus received less objection than gender selection for a female foetus. A higher level of religious observance was significantly associated (P < 0.01) with objection towards egg, sperm, and embryo donation. Female physicians tended to be more supportive of these treatments
Bokek-Cohen, Y., Marey-Sarwan, I., & Tarabeih, M. (2021). Underground gamete donation in Sunni Muslim patients. *Journal of Religion and Health*, 61(4), 2905–2926. 10.1007/s10943-021-01440-1	Qualitative Study	Two Sunni Muslim gynecologists and 25 Sunni Muslim patients who underwent third-party gamete donation treatments. Participants live in two undisclosed Muslim countries	Participants were recruited through the two physicians' gynecological clinics. In-depth in-person interviews took place, during which participants were asked to describe the process they went through and their reactions to the prohibition against third-party gamete donation. Microanalysis of the data was done to detect common themes	In both countries, the physicians facilitating third-party donations had in place complex procedures to receive sperm or egg donations, without placing the donors or recipients at risk. For the patient participants in this study, there was stress and sorrow surrounding the legal prohibitions on third-party ART. Participants displayed subservice attitudes towards religious authorities, and had experienced disapproval when going to religious leaders for guidance. Both sperm or egg recipients reported that their acceptance of a donated gamete was kept secret from friends, family, and their community. In some cases, women had received a sperm donation without their husband's knowledge. There was a desire to protect their husband's image of fertility. Some couples still do not feel they are the true parents if they borrowed sperm or eggs to reproduce
Bokek-Cohen, Y., Marey-Sarwan, I., & Tarabeih, M. (2022). Violating religious prohibitions to preserve family harmony and lineage among Sunni Muslims. *Marriage & Family Review,* 58(3), 245–270. 10.1080/01494929.2021.1953667	Qualitative Study	25 Sunni Muslim women who underwent third-party gamete donation treatments in two undisclosed Middle Eastern countries	This study aimed to identify how infertile women navigate contradicting expectations to adhere to religious prohibitions on third-party gamete donation, on the one hand, and the societal pressures to bear children, on the other. Phenomenological approach was used and interviews were conducted with fertility patients. Data was coded and analyzed using a constant comparison approach	Women chose to undergo illegal third-party gamete donation due to social, familial, marital, and personal pressures/desires to bear children. Participants wished to continue their family line, and there were subversive attitudes towards religious authorities who disapproved of third-party gamete donation. Some participants had receive direct guidance from religious leaders to remain childless rather than accept a donation. Participants kept their procedures a secret from friends, family, and in some cases, even their husbands. Participants wished to preserve their husband's image of virility and masculinity
Bokek-Cohen, Y., & Tarabeih, M. (2022) What do Sunni Muslims think about religiously forbidden reproductive options? *Human Fertility,* 25(4), 764–775. 10.1080/14647273.2021.1921289	Cross-sectional study	824 married Sunni Muslims (381 men and 443 women) in undisclosed locations	Snowball and convenience sampling recruited participants for an online questionnaire collecting data on participants' demographics as well as their levels of approval with regards to ARTs. Significance testing was performed to identify associations between variables	The highest level of objection was directed at sperm donation, followed by sex selection of a female fetus. Respondents also did not accept the option of egg donation and surrogacy. Results turned up no gender differences in levels of acceptance of different types of ART. Higher levels of religious adherence was not significantly correlated with greater disapproval
Clarke, M., & Inhorn, M.C. (2011). Mutuality and immediacy between marja' and muqallid: Evidence from male in vitro fertilization patients in shi'i Lebanon. *International Journal of Middle Eastern Studies.,* 43(3), 409–427. https://www.jstor.org/stable/23017310	Ethnography	Middle Eastern men undergoing infertility treatment, 220 Lebanese, Syrian, and Lebanese-Palestinian. Of the patients, the largest group (seventy-six men, or 35% of the total) identified themselves as Shia	Interviews with patients were carried out by Inhorn in two clinical settings in Beirut: one a major university teaching hospital catering to a religiously mixed population; the other a private clinic, part of a transnational operation headed by a notable Lebanese practitioner and catering primarily, although not exclusively, to Shia Lebanese patients. Interviews involved discussion of which religious leaders participants turned to for guidance	Most participants stated following guidance/rulings from Beirut-based Ayatollah Fadlallah; and the Iranian Ayatollah Khamina'i. A number of the interviewees talked at some length about the religious ethics of the medical procedures they were contemplating and the above religious authorities, towards whom they turned for guidance. Those who followed Fadlallah praised him for being “modern” and “contemporary” and for approaching new issues, technologies, and social phenomena with an “open mind.” To his enthusiasts, Fadlallah’s opinions were seen to be more in harmony with local needs, and this extended to his rulings on assisted reproduction. However, Khamina'i was more supportive of some procedures. For example, sperm donation has faced the stiffest patient opposition, and half of those men who identified al-Khamina'i as their marja disagreed with his position permitting the use of donor sperm. This included even men who were azoospermic. When practitioners recommended certain procedures, participants went to their local Shaykhs for guidance
Gameiro, S., El Refaie, E., de Guevara, B.B., & Payson, Al. (2019). Women from diverse minority or religious backgrounds desire more infertility education and more culturally and personally sensitive fertility care. *Human Reproduction,* 34(9), 1735–1745. 10.1093/humrep/dez156	Qualitative Study	Participants were nine adult women with a minority ethnic or religious status living in Wales, UK, who were experiencing or had experienced infertility in the past. The average age was 42 (range 30–59). Five women were South Asian Muslims, two were Sub-Saharan African Christians, one was a North African Muslim, and one a British Muslim married to a North African Muslim man	A convenience sample was recruited in partnership with a local charity. Participants attended a workshop where visual and textual data about the participants' views and experiences of infertility and their fertility care needs was collected. Thematic analysis was performed	Infertility was an emotional, physical, and social burden. There was high community pressure for parenthood, but low levels of appropriate socio-cultural care. Participants had reservations about certain procedures (i.e. using donated sperm) due to religious beliefs. However, religion was considered a strong comfort during infertility. Some participants believed religious leaders should be engaged more to provide education and support surrounding infertility
Hörbst, V. (2016). 'You cannot do IVF in Africa as in Europe': The making of IVF in Mali and Uganda. *Reproductive BioMedicine and Society Online*, 2, 108–115. 10.1016/j.rbms.2016.07.003	Qualitative Study	Male and female fertility physicians, patients, surrogates, and egg donors at clinics in Muslim-majority Mali and Christian-majority Uganda. Interviews with religious leaders also took place	Interviews, focus groups, and casual conversations were conducted to gather an understanding of participants' experiences of and views on IVF and ART treatments	Some Muslim physicians felt they were going against religious rulings when providing ART using third-party gametes. However, they continued to do so regardless. Muslim patient participants rarely spoke of having consulted Imams for ethical advice, as they mostly believed that questions of the religious ethics of ART were part of private negotiations with God. Both Muslim and Christian male patients were reluctant to use (as a last resort) donor sperm than were women to use donor eggs. These beliefs were related to lineage
Husain, W., & Imran, M. (2020). Infertility as seen by the infertile couples from a collectivist culture. *Journal of Community Psychology*, 49(2), 354–360. 10.1002/jcop.22463	Qualitative Study	20 infertile couples (40 individuals) undergoing IVF and ICSI in clinics/hospitals in Islamabad, Pakistan	Participants were recruited through private fertility clinics and public hospitals. Interviews aimed to gather data on participant demographics and the barriers to fertility treatment they had experienced. Thematic analysis was performed	There were several relevant cultural barriers involved in receiving treatment. These included join-family systems, financial burdens, lack of education on options, as well as treatment contravening religious teachings. There was also stigma around accessing fertility treatment, and distrust of those treatments. However, only 3% reflected that getting infertility treatment is contradictory to the religious teachings
Igbolekwu, C.O., Mkperedem, A.A., Arisukwu, O.C., Uwadinma-Idemudia, E., Iwuh, J., & Olawale, A.A. (2022). Religious and cultural interpretations of artificial insemination in South-West Nigeria. *AJOG Global Reports,* 28(2), Article 100,113. 10.1016/j.xagr.2022.100113	Mixed Methods	493 Muslim and Christians of reproductive age in SW Nigeria, as well as 13 Christian or Muslim cultural/religious leaders	Quantitative data was collected using questionnaires, and qualitative data collected via interviews with cultural/religious leaders. For the questionnaires, 72 participants were randomly selected, and an additional 421 were selected to complete the questionnaire. The questionnaire collected demographic information and views on/knowledge of artificial insemination (AI), particularly with relation to religion. Significance testing (chi-square tests) was performed to draw out associations	Results showed a significant (P < 0.01) relationship between participants' religious denomination and their acceptance of AI. Sunni Muslims were most likely to be accepting of AI (75%), followed by Protestants (66.7%), Pentecostals (65%), and Shi'a Muslims (60%)
Iliyasu Z., Galadanci, H.S., Abubakar, I.S., Bashir, F.M., Salihu, H.M., & Aliyu, M.H. (2013). Perception of infertility and acceptability of assisted reproduction technology in northern Nigeria. *Nigerian Journal of Medicine,* 22(4), 341–347	Cross-sectional Study	300 married couples (600 individuals) residing in Kano, Nigeria. Participants were from Muslim (98.6%) and Christian (1.4%) backgrounds	The survey was descriptive and cross-sectional in design. A multi-stage sampling techniques was used. The questionnaire given to participants involved questions related to demographics, before asking participants about their awareness and acceptability of ART. The questionnaire was validated through comparing results with a separate sample. Data was analyzed first categorically, then through bivariate analysis. Significance testing (Chi-square tests) was performed	Only 7.6% of respondents were willing to accept ART. By sex, more male respondents (11.0% versus 4.1%) were accepting. Of the acceptors, most (61.4%) preferred 'test tube' baby, 34.1% chose surrogate motherhood and only a few (4.5%) would accept AI. The reasons for rejecting ART included anxieties over parent–child bonding (86.2%), guilt related to payment for surrogacy (80.2%), concerns about genetic abnormalities (61.8), and doubt over religious permissibility (53.2). A higher proportion of non-Muslims were willing to accept ART
Inhorn, M.C. (2006a). Making Muslim babies: IVF and gamete donation in Sunni versus Shi'a Islam. *Culture, Medicine and Psychology,* 30(4), 427–50. 10.1007/s11013-006-9027-x	Ethnography	500 + infertile Muslim IVF patients, both men and women, in Egypt and Lebanon	This ethnographic research draws on interviews with infertile couples over several years, placing them in the context of Sunni and Shi'a religious and legal frameworks for understanding and accepting ARTs	The majority of participants (both Sunni and Shi'a Muslims) believed that gamete donation is religiously forbidden. When couples are unable to have biological children of their own—with or without the support of ARTs—many choose to stay together without children. Divorce is not an immediate consequence of infertility. Because of the Sunni Islamic restrictions on the use of donor eggs, however, as well as lack of acceptance of this option among some segments of the Shi’ite population, at least some Muslim men are choosing to divorce or take a second wife. Some couples/individuals still choose to take a donor gamete even if it is considered religiously forbidden
Inhorn, M.C. (2006b). "He won't be my son": Middle Eastern Muslim men's discourses of adoption and gamete donation. *Medical Anthropology Quarterly,* 20(1), 94–120. 10.1525/maq.2006.20.1.94	Ethnography	200 + Lebanese, Syrian, and Palestinian men residing in Lebanon or expatriates of Lebanon. Approximately half of the men were infertile, and the other half were infertile controls (i.e. the husbands of infertile women)	Participants were recruited through IVF clinics during their treatment. Interviews were conducted to assess men's attitudes towards IVF and particularly third-party gamete donation. 32% of participants were Sunni Muslims, 36% were Shi'a Muslims, 26% were Christian, and 4% were Druze	No matter the religious sect, most Muslim men in Lebanon continue to resist both adoption and gamete donation, arguing that such a child "won't be my son." Sunni Muslims were more disapproving (83%), compared to Shi'a Muslims (64%). However, some Muslim men are considering and undertaking these alternatives to family formation as ways to preserve their marriages, satisfy their fatherhood desires, and challenge religious dictates, which they view as out of step with new developments in science and technology
Inhorn, M.C. (2004). Middle Eastern masculinities in the age of new reproductive technologies: male infertility and stigma in Egypt and Lebanon. *Medical Anthropology Quarterly*, 18(2),162–82. 10.1525/maq.2004.18.2.162	Ethnography	66 couples (132 individuals) receiving treatment for infertility in IVF clinics in Cairo, Egypt. 70% of the couples were experiencing male-factor infertility. In addition, 220 Lebanese, Syrian, and Palestinian men were recruited from Beirut, Lebanon. 120 were experiencing infertility, and 100 were the husbands of a woman experiencing infertility	In Cairo, both husbands and wives participated in interviews, which involved discussion of a wife variety of issues pertaining to IVF, male infertility, and treatments. In Lebanon, interviews were conducted with the men only. Participants were asked about concepts of masculinity, marriage, morals, and modern treatments	Male infertility was considered an emasculating experience. Few men in Cairo were willing to tell anyone that they were suffering from infertility. However, male infertility was more accepted as a medical problem in Lebanon. Infertile men were worried about the stigma that might surround their IVF child, due to a popular assumption that IVF children always involve donor gametes. Some believed that IVF was forbidden. However, most believed that new technologies—namely ICSI—brought new hope for them
Inhorn, M. C. (2007). Masturbation, semen collection and men’s IVF experiences: Anxieties in the Muslim world. *Body & Society,* 13(3), 37–53. 10.1177/1357034X07082251	Ethnography	250 patients receiving infertility treatment in IVF clinics in Egypt and Lebanon	Ethnographic research was conducted in Egypt and Lebanon. The research examined what happens for men who refuse such masturbation – either on religious grounds or by virtue of psychological trauma – as well as what happens to men who fail to produce the imperative semen sample. Interviews were conducted with male patients experiencing infertility themselves, or whose wives were experiencing infertility. Research also involved an analysis of the physical clinical spaces required for semen collection, and their appropriateness for Muslim men	For Muslim men, the IVF experience takes on additional complex meanings of sin, guilt and even illicit pleasure. There, masturbation connotes illicit sexuality, and is deemed by some men to be the cause of their own male infertility. Furthermore, semen, though life-giving, is also deemed polluting, a source of impurity which requires ablution before prayer. Given these ambivalences and ambiguities, Muslim men in Middle Eastern IVF clinics may be especially conflicted about delivering semen samples in the clinic. Furthermore, clinic practices may either exacerbate men’s anxieties, when sexual privacy cannot be guaranteed, or promote guilty pleasures, when illicit pornography is made available as a mechanism of sexual stimulation
Isikoglu, M., Senol, Y., Berkkanoglu, M., Ozgur, K., Donmez, L., & Stones-Abbasi, A. (2006). Public opinion regarding oocyte donation in Turkey: first data from a secular population among the Islamic world. *Human Reproduction,* 21(1),318–323. 10.1093/humrep/dei274	Cross-sectional study	400 Muslim residents of two undisclosed Turkish cities	A cluster sampling method was employed to recruit participants to fill in a questionnaire. The questionnaire collected demographic data, before asking participants about their knowledge on oocyte donation and attitudes towards it. χ2- and trend χ2-tests were used for the statistical analyses of the data and α < 0.05 was considered statistically significant	Most participants reported that if they had a child by oocyte donation they would have told their friends and relatives. More than half of the women and nearly two-thirds of the men thought that their religious would allow oocyte donation. More men than women approved of oocyte donation (P < 0.05). Only 15.25% of participants showed complete objection to oocyte donation. The vast majority of the respondents thought that if people needed oocyte donation, knowledge of the treatment should be restricted to the man, the woman and the doctor and that the child should never be informed
Lestari, A.Y., Jubba, H., Jenie, S.I., Iribaram, S., & Adawiah, R. (2022). Young Muslims’ responses to conception through in-vitro fertilization. *Cogent Social Sciences,* 8(1), Article e2076323. 10.1080/23311886.2022.2076323	Mixed Methods	566 students and 8 faculty members from one university in Yogyakarta, Indonesia	Data was collected through questionnaires, interviews, and document reviews. Student questionnaires collected data on participant demographics, as well as their beliefs surrounding the permissibility and acceptability of married couples' use of in-vitro fertilization. Interviews were conducted with academic staff on the same topic. The data were analyzed using a progressive constructivist approach	85.7% of student participants approved of married couples' use of IVF for contraception. Most participants (72.79%) believed IVF was permitted by religious law. Respondents emphasized that IVF is not intended simply to facilitate conception; it is also intended to continue the bloodline, ensure the family’s happiness, and maintain the honor of the married couple, per the teachings of Islam. For those who did not approve of IVF, this disapproval was based around religious beliefs in that IVF was going against religiously-dictated fate
Martin-Anatias, N., & Davies, S.G. (2023). Children as investment: Religion, money, and Muslim migrants' experiences of assisted reproduction in Aotearoa New Zealand (NZ). *Journal of Cross-Cultural Gerontology*, 38(4), 307–325. 10.1007/s10823-023-09491-5	Qualitative Study	18 migrant Muslim women from Indonesian, Sri Lankan, Malay Singaporean, and Indian backgrounds experiencing infertility. All participants were living in Aotearoa New Zealand	Semi-structured interviews were performed to gather information on participants' experiences of infertility and associated treatments, in light of cultural and religious contexts. The data was analyzed thematically	Participants wished to conceive in a religiously-permissible way. The research participants understood that IVF was a controversial issue amongst many Muslims. Despite this awareness, several participants stated they actively ignored Islamic ideals out of desperation to conceive. Other participants sought out knowledge surrounding the permissibility of IVF in Islam through their religious communities and online. They relied heavily on their networks' knowledge, and assume that IVF is permissible as their community members in NZ and in their home countries are participating in the treatment. Some set religious boundaries on what was and was not permissible for them. e.g., IVF was acceptable with their own material, but not when it included donated gametes. Children were seen as a religious investment, and central to the practice of their faith
Monroe, K.V. (2022): Planning for the family in Qatar: Religion, ethics, and the politics of assisted reproduction. *Ethnos.* 10.1080/00141844.2022.2057563	Ethnography	15 (8 men and two women) fertility specialists (physicians, lab directors, prenatal genetic counsellors) practicing in Doha, Qatar	Participants were recruited from six IVF clinics in Doha. One-on-one and group interviews were conducted to gather information on participants' professional backgrounds, their experiences working with ARTs in Qatar, and the ways Muslim patients experience and negotiate ARTs	Clinicians worked within a setting where they were often having conversations around what is right, what is wrong, and what is possible. Participants related that the first question that many patients have when they mention IVF was whether or not it is allowed according to Islamic law. Patients were also concerned about gametes being 'mixed up' in the laboratory. Participants used religion to reassure patients about procedures. Although third-party donation was illegal in Qatar, participants still told patients that these options exist elsewhere, as they viewed it as the patients' responsibility to determine for themselves whether an option is ethically acceptable or not
Murad, Z.A., Shah, Y.Z., Mansor, S., Ahmad Irfan, I.H., & Abdullah, L. (2014). Is assisted reproductive technique shari’aa-compliant? A case study at a fertility centre in Malaysia. *International Medical Journal Malaysia.* 13(2), 21–27. 10.31436/imjm.v13i2.473	Qualitative Study	Participants comprised 21 patients, 2 embryologists, 2 doctors, 4 paramedics, and 1 counsellor in Kuantan, Malaysia	Participants were recruited from the fertility clinic and provided with a questionnaire. The questionnaire collected data on participant demographics as well as their experiences accessing/providing fertility treatments. Namely, whether they faced any concerns as a Muslim accessing/providing this treatment. The data was qualitatively analyzed according to themes	Participants had many concerns related to the religious permissibility of ARTs. These included things like, the conditions under which it is permissible, whether masturbation to derive samples are allowed, whether patients were permitted to receive treatment from a practitioner of the opposite sex, whether ghusul (ritual washing) was required after receiving treatment, and whether third-party gamete donations were acceptable. Participants were often familiar with some expert’s fatwas pertaining to ART and IVF in Malaysia
Reaves, S.N., & Hauck, F.R. (2019). Infertility in Muslim refugees: A review of the literature. *Journal of Refugee & Global Health*. 2(2), Article 9. 10.18297/rgh/vol2/iss2/9	Literature review	Muslim immigrants and/or refugees experiencing infertility and living in Middle Eastern countries	Articles were included that focused on the use of assisted reproductive technology among Muslim patients from the Middle East, the impact of infertility among Muslim men and/or women from the Middle East, and barriers to care among immigrants and/or refugees in accessing health care for ART	A total of 37 articles met the inclusion criteria and were reviewed. There were several common themes across the literature: Islamic perspectives on ART, the psychosocial impacts of infertility, and barriers to treatment. Muslim men reported feelings of emasculation and some blamed themselves for their infertility, believing it was a punishment for engaging in premarital sex or that they had “spent” all of their Sperm. Women from Middle Eastern countries reported feeling significant societal pressure to bear children. In cases of infertility, women may be blamed for “reproductive failing” which may lead to marital discord Barriers to diagnosis and management of infertility for immigrants and refugees include the high cost of ART, language and cultural barriers, and difficulty navigating a new health care system
Salehi, K., Shakour, M., Pashaei Sabet, F., & Alizadeh, S. (2015). The opinion of Iranian students about the society's perception on using surrogacy as an infertility treatment in the future community. *Sexual and Reproductive Healthcare*, 6(1),19–22. 10.1016/j.srhc.2014.06.005	Cross-sectional Study	200 students (23 male and 147 female) of Isfahal University, Iran in the following courses: Midwifery, medicine, psychology, and law	Quota sampling method was used to recruit a study population. A questionnaire was provided to participants which included questions on their knowledge on, attitude towards, and acceptance of surrogacy as a solution to infertility. Significance testing (Chi-square tests) were performed to identify associations between variables	The vast majority of participants agreed with surrogacy as a viable method of parenthood for infertile couples. Students of the medical course were mostly in the category “strongly agree” and “agree” with surrogacy (79.6%), then midwifery students (78.9%). Psychology and law students also largely agreed/strongly agreed with this method (74.5 and 79.4 respectively), though also contained a larger proportion of participants who disagreed or were conservative on the topic. According to chi-square test, there was no significant difference between attitudes of students (P = 0.08)
Scully, J.L., Banks, S. Song, R., & Haq, J. (2017). Experiences of faith group members using new reproductive and genetic technologies: A qualitative interview study. *Human Fertility*. 20(1), 22–29. 10.1080/14647273.2016.1243816	Qualitative Study	16 participants (13 Christian, 3 Muslim) with experience of new reproductive and genetic technologies (NRGT) living in the UK	In-depth interviews were performed. Interviews elicited information about participants' experiences with NRGTs, the ethical and religious factors impacting their experiences, and whether they had experienced guidance from a faith counsellor. Themes in the data were identified through close reading and inductive thematic analysis	Faith issues and considerations were not raised or addressed by practitioners in the fertility healthcare setting. One Muslim participant questioned the religious permissibility of egg donation. Participants believed that healthcare professionals were insensitive or disinterested in patients' religious considerations due to lack of time, work pressures, and fear that it would be inappropriate to raise these topics. Both Christian and Muslim participants were unclear about the official teachings of their respective sects/faiths. They also found it difficult to find information on official positions
